# Unravelling Different Water Management Strategies in Three Olive Cultivars: The Role of Osmoprotectants, Proteins, and Wood Properties

**DOI:** 10.3390/ijms252011059

**Published:** 2024-10-15

**Authors:** Sara Parri, Claudia Faleri, Marco Romi, José C. del Río, Jorge Rencoret, Maria Celeste Pereira Dias, Sara Anichini, Claudio Cantini, Giampiero Cai

**Affiliations:** 1 Department of Life Sciences, University of Siena, Via Mattioli 4, 53100 Siena, Italy; sara.parri2@unisi.it (S.P.); claudia.faleri2@unisi.it (C.F.); romi5@unisi.it (M.R.); sara.anichini@unifi.it (S.A.); 2 Instituto de Recursos Naturales y Agrobiología de Sevilla, CSIC, Reina Mercedes 10, E-41012 Seville, Spain; delrio@irnase.csic.es (J.C.d.R.); jrencoret@irnase.csic.es (J.R.); 3 Centre for Functional Ecology, Department of Life Sciences, University of Coimbra, Calçada Martim de Freitas, 3000-456 Coimbra, Portugal; celeste.dias@uc.pt; 4 Department of Agriculture, Food, Environment and Forestry, University of Florence, Piazzale delle Cascine, 18, 50144 Firenze, Italy; 5 Institute for BioEconomy (IBE), National Research Council (CNR), Strada Provinciale Aurelia Vecchia 49, 58022 Follonica, Italy; claudio.cantini@ibe.cnr.it

**Keywords:** *Olea europaea*, osmoprotectants, Western blotting, wood anatomy, sugars, 2D-NMR spectroscopy, lignin, dehydrins, osmotin, aquaporins

## Abstract

Understanding the responses of olive trees to drought stress is crucial for improving cultivation and developing drought-tolerant varieties. Water transport and storage within the plant is a key factor in drought-tolerance strategies. Water management can be based on a variety of factors such as stomatal control, osmoprotectant molecules, proteins and wood properties. The aim of the study was to evaluate the water management strategy under drought stress from an anatomical and biochemical point of view in three young Italian olive cultivars (Giarraffa, Leccino and Maurino) previously distinguished for their physiological and metabolomic responses. For each cultivar, 15 individuals in pots were exposed or not to 28 days of water withholding. Every 7 days, the content of sugars (including mannitol), proline, aquaporins, osmotins, and dehydrins, in leaves and stems, as well as the chemical and anatomical characteristics of the wood of the three cultivars, were analyzed. ‘Giarraffa’ reduced glucose levels and increased mannitol production, while ‘Leccino’ accumulated more proline. Both ‘Leccino’ and ‘Maurino’ increased sucrose and aquaporin levels, possibly due to their ability to remove embolisms. ‘Maurino’ and ‘Leccino’ accumulated more dehydrins and osmotins. While neither genotype nor stress affected wood chemistry, ‘Maurino’ had a higher vessel-to-xylem area ratio and a larger hydraulic diameter, which allows it to maintain a high transpiration rate but may make it more susceptible to cavitation. The results emphasized the need for an integrated approach, highlighting the importance of the relative timing and sequence of each parameter analyzed, allowing, overall, to define a “strategy” rather than a “response” to drought of each cultivar.

## 1. Introduction

Olive production is crucial for the economy, agriculture and biodiversity of the Mediterranean region. However, climate change is causing significant warming and unpredictable rainfall, leading to more frequent and severe droughts that affect agriculture [1]. Olive trees are highly sensitive to changes in local microclimatic conditions [2], resulting in reduced photosynthetic performance, oxidative stress, and nutrient imbalances [3]. Understanding the response of olive trees to drought stress is essential for improving cultivation practices, developing drought-tolerant cultivars, enhancing long-term water management, and preserving olive oil quality.

Olive trees exhibit a multifaceted response to drought, involving morphological, physiological, genetic and biochemical changes that have been extensively studied in leaves [4]. However, the stem plays an important role in plant growth, water transport, carbohydrate and water storage [5]. Water management within plants also depends on factors such as stomatal control, osmoprotectant molecules [6], and chemical and anatomical properties of wood [7]. These factors are interrelated, as wood properties affect water transport along the stem, which can be affected by drought and subsequently carbon availability [8]. The most common molecules involved in water management in plant tissues are osmoprotectants, such as sugars, including mannitol, and proline [9,10].

During water shortage, the accumulation of these compounds lowers the water potential of leaf cells and increases their tolerance to water deficit [11]. In two-year-old olive plants exposed to drought stress, the levels of soluble carbohydrates such as mannitol and glucose increased depending on cultivar, stress intensity and duration [12,13].

Mannitol, a solute found in algae and plants, improves drought tolerance in olive trees by a cultivar-dependent increase under moderate stress [13]. Mannitol levels were also increased in olive plants exposed to drought and treated with ameliorants [14]. The Tunisian cultivar Picholine showed higher glucose and mannitol levels under strict irrigation regimes [10], and, in general, mannitol has been identified as the key carbohydrate involved in water stress resistance [11]. Comparison of drought-resistant olive cultivars (Chemlali and Meski) revealed that ‘Chemlali’ accumulated mannitol and phenolic compounds while increasing antioxidant enzyme activity [15]. Olive trees under drought expressed stress-related genes that promote starch breakdown and mannitol production [16]. Two-year-old olive trees under water deficit showed decreased leaf water potential, relative water content, and osmotic potential, with mannitol levels increasing in water-stressed tissues [17]. In addition, olive plants responded to drought and salt stress by increasing the activity of the mannitol transporter [18,19].

Proline enhances drought tolerance in plants by balancing water levels, scavenging reactive oxygen species, and regulating stress-responsive gene expression [20]. In olive plants, drought stress increases proline, malondialdehyde and lipoxygenase levels, while decreasing transpiration, photosynthetic rate, stomatal conductance and CO_2_ concentration [21]. Water deficiency reduced shoot elongation and photosynthetic performance in Tunisian olive cultivars, except for the Chemlali cultivar, which had stronger antioxidant defense mechanisms and more proline content [9]. The drought-stressed olive cultivars Zalmati and Chemlali accumulated more proline [22], while the Mediterranean cultivar Manzanilla, which had higher proline content, outperformed other olive cultivars in terms of photosynthetic rate and drought tolerance [23]. Among several, the drought-stressed Persian and Greek olive cultivars, ‘Dezful’, ‘Amigdalolia’ and ‘Conservolia’ showed higher leaf water content, membrane stability, and proline content [12]. However, proline was not always associated with better physiological performance under drought stress, as both Empeltre and Arbequina cultivars resulted in reduced photosynthesis and vegetative growth, but both showed increased proline [24].

Proteins such as dehydrins, osmotins and aquaporins, may also be involved in drought response by influencing water movement within plants [25]. Dehydrins, also known as group II late embryogenesis abundant (LEA) proteins, are hydrophilic stress proteins produced in response to cold and drought stress [26], presumably through the induction of abscisic acid (ABA) [27]. Research on antioxidant enzymes and proteins in olive trees found that immunologically related dehydrins of 43 and 23 kDa accumulate during cold acclimation, with 43 kDa dehydrin associated with cold hardiness across all cultivars, and the 23 kDa dehydrin being cultivar-dependent [28]. Additionally, a study on drought response genes found that dehydrin expression differed among cultivars [29].

Osmotin, a PR-5 family defense protein, is essential for plant survival under both biotic and abiotic stress conditions [30], acting as an osmoprotectant and providing enzyme protection and protein chaperone functions. In soybean, a novel putative osmotin has been found in the response to drought stress [31]. In barley, osmotin expression increased drought tolerance and improved the enzymatic activity of ascorbate peroxidase [32]. Expression of the osmotin gene may confer resistance to water stress in olive trees, as olive plants expressing the tobacco osmotin gene showed increased tolerance to cold stress [33]. Another study confirmed the protective role of osmotin in cold-tolerant olive varieties, showing a positive correlation between osmotin expression and cold resistance [34], and shoots from Canino cultivar expressing a tobacco osmotin transgene showed higher proline levels and lower lipid peroxidation following drought stress [35].

Aquaporins are transmembrane channel proteins that regulate plant–water interactions [36]. Among the five families of aquaporins, the plasma membrane intrinsic proteins (PIPs), which support long-term water transport, are among those that respond to drought stress [37]. Aquaporin levels were strongly correlated with stomatal and mesophyll conductance in water-stressed olive plants [38]. In Leccino plants, expression of aquaporin-encoding genes decreased with increasing stress, suggesting reduced expression is a response to drought. Analysis of a single aquaporin gene in drought-stressed olive plants gave similar results [39]. However, aquaporin levels increased in olive plants recovering from drought, indicating that reduced aquaporin expression is part of the drought response and that increased expression is necessary for drought recovery [40].

Water transport through xylem vessels and tracheids can be affected by drought-induced cavitation [41], and cavitation susceptibility is critical to plant drought stress resistance, which is influenced by wood anatomical and chemical properties. Higher wood density is associated with greater strength [42,43], but optimal xylem hydraulic properties for drought resistance are still unclear [44,45,46]. Lignin increases cell wall strength, stiffness and hydrophobicity, which helps wood withstand water pressure [47], and higher lignin concentrations correlate with lower susceptibility to embolism [5]. Drought can increase lignin content by promoting its deposition in vessels [47]. The ratio of different lignin units also affects wood properties; in particular, a higher lignin S/G ratio is associated with increased resistance to embolism, as S-rich lignin is more flexible and hydrophilic [5]. In olive wood, vessels are evenly distributed along the annual ring, with small diameter vessels increasing resistance to drought-induced cavitation. Different olive cultivars are likely to have different vessel diameters, which may affect water management [48,49,50].

Previous studies on three Italian olive cultivars (Giarraffa, Leccino and Maurino) showed different responses to drought stress. ‘Giarraffa’ exhibited greater water conservation and earlier stomatal closure, while ‘Leccino’ maintained photosystem II efficiency and low oxidative stress. In contrast, ‘Maurino’ had the highest transpiration rate and experienced increased electrolyte leakage [51]. Metabolomic analyses further revealed cultivar-specific differences, with ‘Maurino’ accumulating higher levels of phenolic compounds in its leaves and stem compared to ‘Leccino’ and ‘Giarraffa’, which instead accumulated epicuticular wax-related compounds [52]. Given these responses, we hypothesized that ‘Leccino’, despite having lower antioxidant pools [53] and higher stomatal conductance than ‘Giarraffa’, may rely on osmoprotectants to retain water in plant tissues. Conversely, ‘Maurino’, with its higher stomatal density and conductance, coupled with lower stem and leaf water content under drought conditions, may be more susceptible to drought-induced cavitation; ‘Giarraffa’ may rely on reduced carbon availability due to reduced gas exchange. To elucidate the underlying mechanisms, this study investigated the role of the PIP1 family aquaporins, osmotins, dehydrins, sugars (including mannitol) and proline in the drought response of these three cultivars. By examining both leaves and stems, the study aimed to understand how biochemical and structural aspects affect water management in different plant organs, and provide a more comprehensive understanding of the drought tolerance of these olive cultivars. 

## 2. Results

### 2.1. Sugar Levels

**Figure 1:**
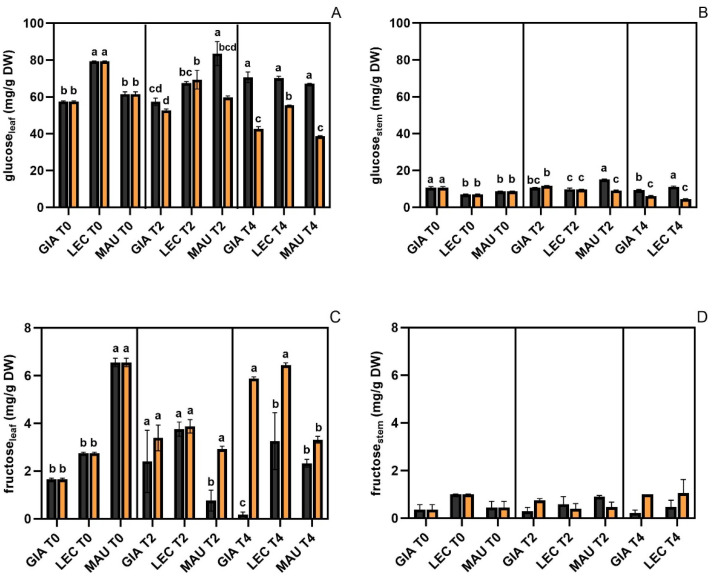

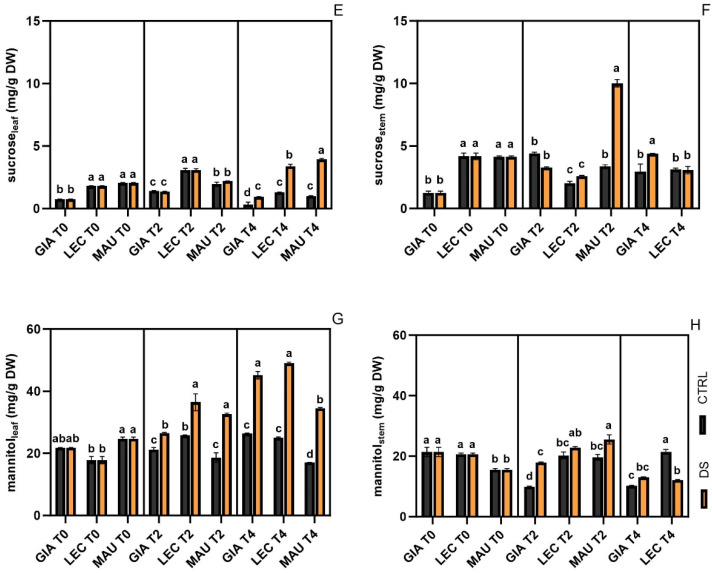
Sugar levels identified by HPLC in Giarraffa (GIA), Leccino (LEC) and Maurino (MAU) under control (CTRL, black) and drought stress (DS, orange). (**A**) Glucose in leaf; (**B**) glucose in stem; (**C**) fructose in leaf; (**D**) fructose in stem; (**E**) sucrose in leaf; (**F**) sucrose in stem; (**G**) mannitol in leaf; (**H**) mannitol in stem, all expressed in mg g^−1^ tissue dry weight (DW). Data in each column are presented as mean ± standard error. Within each time point, different letters denote statistical significance (*p*-value < 0.05) according to Tukey’s multiple post hoc tests.

The glucose levels in the leaves (Figure 1A), which ranged from 40 to 80 mg/g DW, were higher than those found in the stems (Figure 1B), which ranged from 5 to 15 mg/g DW. However, in both leaves and stems, the drought-stressed groups of all cultivars were lower and significantly different from their respective controls at t4. A similar decrease in glucose was observed in the stressed groups of ‘Maurino’ already at t2, both in stems and leaves. Similar to glucose, fructose levels were higher in leaves (Figure 1C) than in stems (Figure 1D). Fructose content in leaves ranged from 1 to 6 mg/g DW, while in stems it varied slightly, around 1 mg/g DW. In stems, no significant differences were found between control and stressed samples for any cultivar. In leaves, fructose levels were higher and significantly different from the controls in the stressed groups of ‘Giarraffa’ and ‘Leccino’ only at t4. At t2, a significant difference between control and stressed samples was found in ‘Maurino’, but the content of the control group was lower compared to all the other experimental groups at the same time point. The sucrose content in leaves (Figure 1E) was consistently below 5 mg/g DW, while in stems (Figure 1F) it varied from 1 to 10 mg/g DW. For all three cultivars, the stressed groups showed a higher and significantly different leaf sucrose content compared to their respective controls, at only t4. However, the accumulation was lower in ‘Giarraffa’ than in ‘Leccino’ and ‘Maurino’. In stems, a significant difference between control and drought-stressed groups was found only in ‘Maurino’ at t2 and in ‘Giarraffa’ at t4. Mannitol in leaves (Figure 1G) was higher in the stressed samples of all cultivars compared to their controls, both at t2 and at t4, and it reached its maximum values above 40 mg/g DW in the DS groups of ‘Giarraffa’ and ‘Leccino’. In the stems (Figure 1H), ‘Giarraffa’ and ‘Maurino’ showed an increase in mannitol under stress conditions at t2, which was higher and significantly different compared to the value of their respective controls. On the contrary, mannitol was lower in the DS group of ‘Leccino’ at t4, compared to the controls.

### 2.2. Proline Content

**Figure 2:**
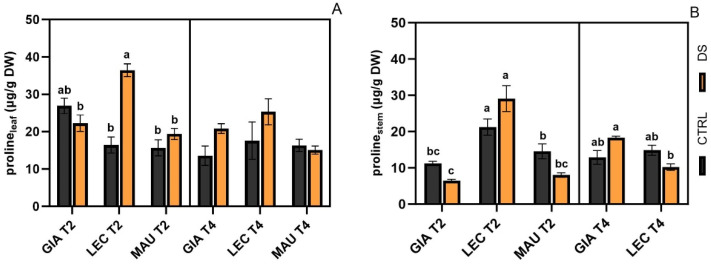
Proline content in leaves (**A**) and stems (**B**) of Giarraffa (GIA), Leccino (LEC), and Maurino (MAU) cultivars under control (CTRL, black) and drought stress (DS, orange). Contents are expressed as μg g^−1^ tissue dry weight (DW). Values in each column are presented as mean ± standard error. Within each time point, different letters denote statistical significance (*p*-value < 0.05) according to Tukey’s multiple post hoc tests.

All cultivars showed similar levels of proline in both leaves (Figure 2A) and stems (Figure 2B). The only exception was the drought-stressed group of ‘Leccino’ at t2, since it had a higher value compared to the other experimental groups and was significantly different from the respective control only in leaves.

### 2.3. Starch Content

The control and drought-stressed groups exhibited nearly equal starch levels across all cultivars, except for ‘Leccino’ and ‘Maurino’ leaves collected early in the morning, where the drought-stressed groups had significantly lower starch levels than the controls. Overall, starch content decreased over time, particularly in stems and leaves collected at dawn, likely due to unfavorable environmental conditions for starch production. Consequently, starch content was not further investigated; the plots can be found in the Supplementary Material (Figure S1).

### 2.4. PIP1 Aquaporin Levels

**Figure 3:**
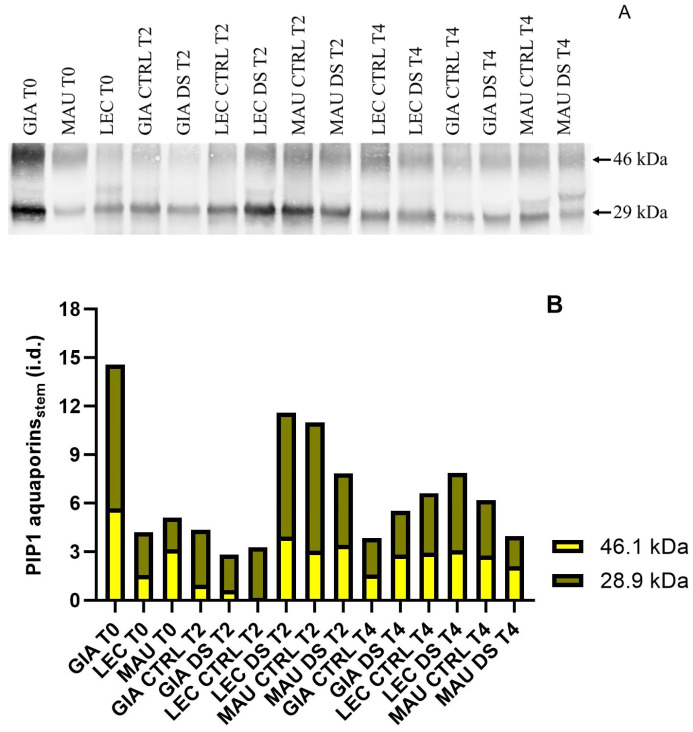
PIP1 aquaporin levels in stems of Giarraffa (GIA), Leccino (LEC) and Maurino (MAU) cultivars under control (CTRL) and drought-stress (DS) conditions, at the beginning of stress (t0), two weeks later (t2) and four weeks later (t4). (**A**) Membranes immunoblotted with anti-aquaporin antibodies from the above experimental groups; (**B**) relative blot quantification expressed as integrated density (i.d.).

Blots relative to aquaporins revealed three or more bands, but only the two most distinct bands (46 and 29 kDa) were quantified (Figure 3A). Densitometric analysis (Figure 3B) showed significant variation in protein concentrations among the experimental groups. At t0, ‘Giarraffa’ exhibited higher PIPs1 content than the other cultivars, but this content decreased at later time points. In stressed stem samples, PIPs1 accumulation in ‘Giarraffa’ was lower at t2 but higher at t4 compared to controls. The stressed groups of the ‘Leccino’ cultivar accumulated significantly more PIPs1 than the controls at both t2 and t4. Conversely, ‘Maurino’ accumulated fewer PIPs1 in the stressed groups than in the controls at both time points.

### 2.5. Dehydrin Levels

**Figure 4:**
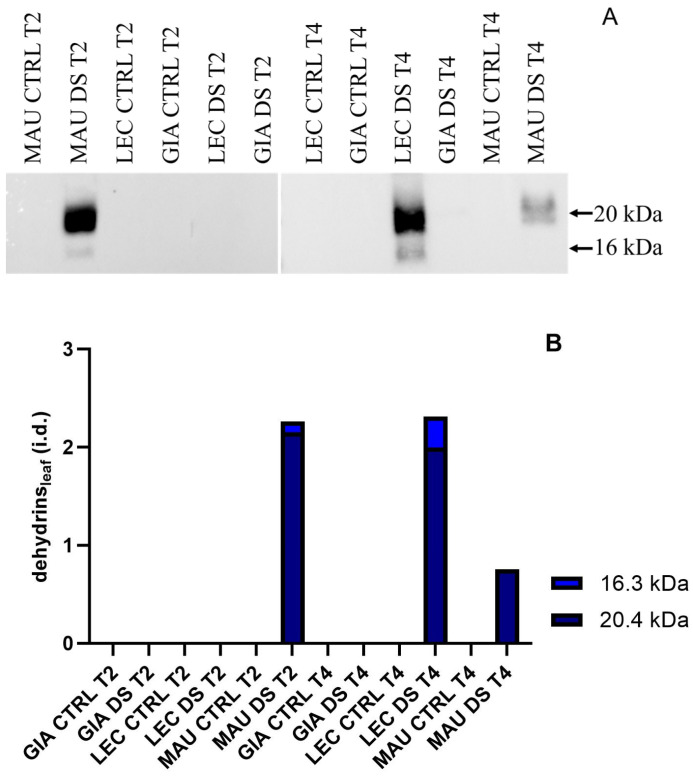
Dehydrin levels in leaves of Giarraffa (GIA), Leccino (LEC) and Maurino (MAU) cultivars after two (t2) and four (t4) weeks of stress. (**A**) Membranes immunoblotted with anti-dehydrin antibodies from the above experimental groups; (**B**) relative quantification of the blots expressed as integrated density (i.d.).

Figure 4 shows dehydrin blots on leaves, with equal amounts of total protein per lane for all experimental groups. Two polypeptides, with molecular weights of 20 and 16 kDa, were identified and quantified (Figure 4A). The results in Figure 4B clearly indicate that only two of the three cultivars contained dehydrins, both from drought-stressed groups: Maurino DS exhibited a significant increase in dehydrins at t2, which remained present at lower levels at t4. The stress group of ‘Leccino’ did not show significant dehydrin accumulation until late in t4. No dehydrins were detected in the Giarraffa cultivar.

**Figure 5:**
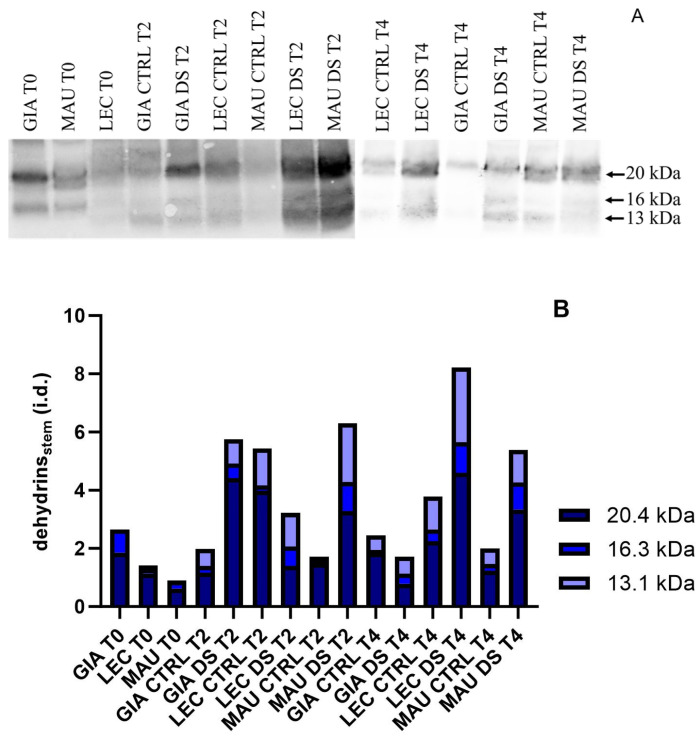
Dehydrin levels in stems of Giarraffa (GIA), Leccino (LEC) and Maurino (MAU) cultivars under control (CTRL) and drought-stress (DS) conditions, at the beginning of stress (t0), two weeks later (t2) and four weeks later (t4). (**A**) Membranes immunoblotted with anti-dehydrin antibodies from the above experimental groups; (**B**) relative blotting quantification expressed as integrated density (i.d.).

From the immunological assay of the stem, three polypeptides (molecular weights 13, 16, and 20 kDa) were selected for measurement (Figure 5A). Subsequent densitometric analysis (Figure 5B) showed that only the cultivar Maurino accumulated these proteins in drought-stressed groups at t2 and t4. ‘Giarraffa’ and ‘Leccino’ exhibited opposing patterns, with ‘Giarraffa’ DS showing high levels of dehydrins at t2 but a moderate reduction at t4, compared to the controls. In contrast, ‘Leccino’ DS decreased at t2 compared to controls, but increased significantly by t4.

### 2.6. Osmotin Levels 

**Figure 6:**
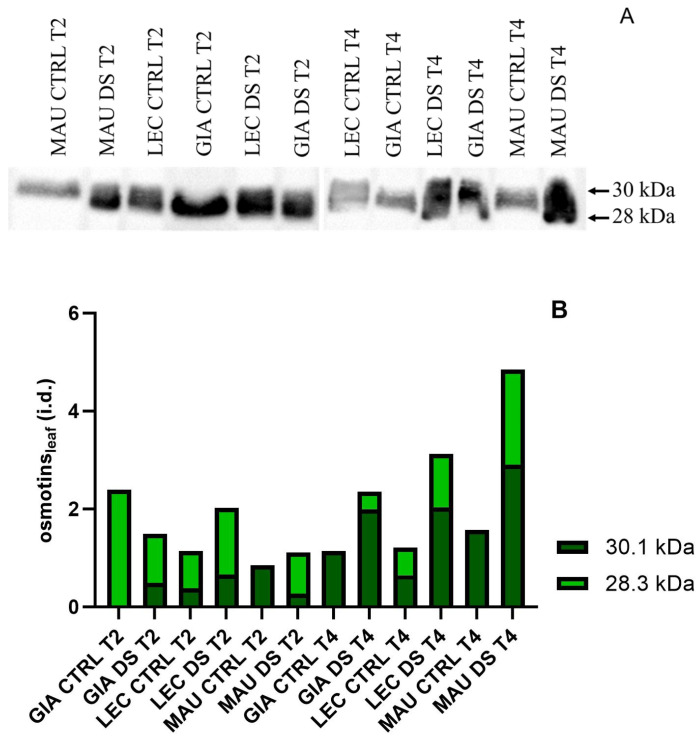
Osmotin levels in leaves of Giarraffa (GIA), Leccino (LEC) and Maurino (MAU) cultivars after two (t2) and four (t4) weeks of stress. (**A**) Membranes immunoblotted with anti-osmotin antibodies from the above experimental groups; (**B**) relative quantification of blotting expressed as integrated density (i.d.).

Figure 6 shows osmotin blots on leaves. Two isoforms, at 30 and 28 kDa, were identified (Figure 6A), although they were difficult to distinguish. As shown by the relative quantification in Figure 6B, at t4 osmotin was highly accumulated in the stressed groups of all cultivars, with ‘Maurino’ DS showing the highest accumulation. At t2, ‘Giarraffa’ DS showed lower osmotin levels than the control group, in contrast to ‘Leccino’ and ‘Maurino’.

**Figure 7:**
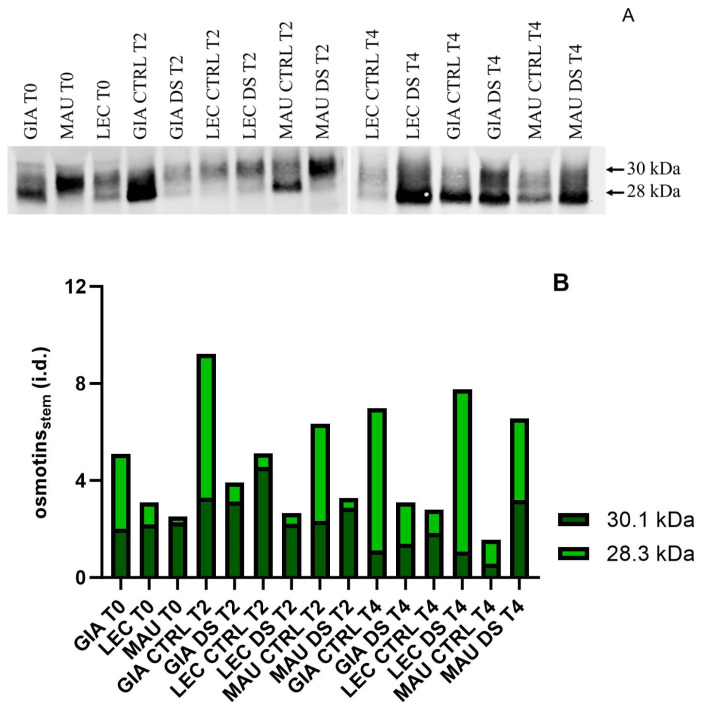
Osmotin levels in stems of Giarraffa (GIA), Leccino (LEC) and Maurino (MAU) cultivars under control (CTRL) and drought-stress (DS) conditions, at the beginning of stress (t0), two weeks (t2) and four weeks (t4). (**A**) Membranes immunoblotted with anti-osmotin antibodies from the above experimental groups; (**B**) relative blotting quantification expressed as integrated density (i.d.).

In stems, two bands, at 30 kDa and 28 kDa, were consistently detected (Figure 7A). The relative levels (Figure 7B) showed that all cultivars reduced the accumulation of osmotin at t2. While ‘Giarraffa’ DS maintained a low level of osmotin also at t4, osmotin accumulation increased in ‘Maurino’ DS and ‘Leccino’ DS.

### 2.7. Lignin Content and Composition

The lignin content analyzed in the stems of ‘Giarraffa’, ‘Leccino’, and ‘Maurino’ at t4 was similar across the cultivars, regardless of the treatment (Giarraffa: 23.3 ± 0.1%; Leccino: 23.0 ± 0.1%; Maurino: 22.7 ± 0.2% of total wood weight). 

**Figure 8:**
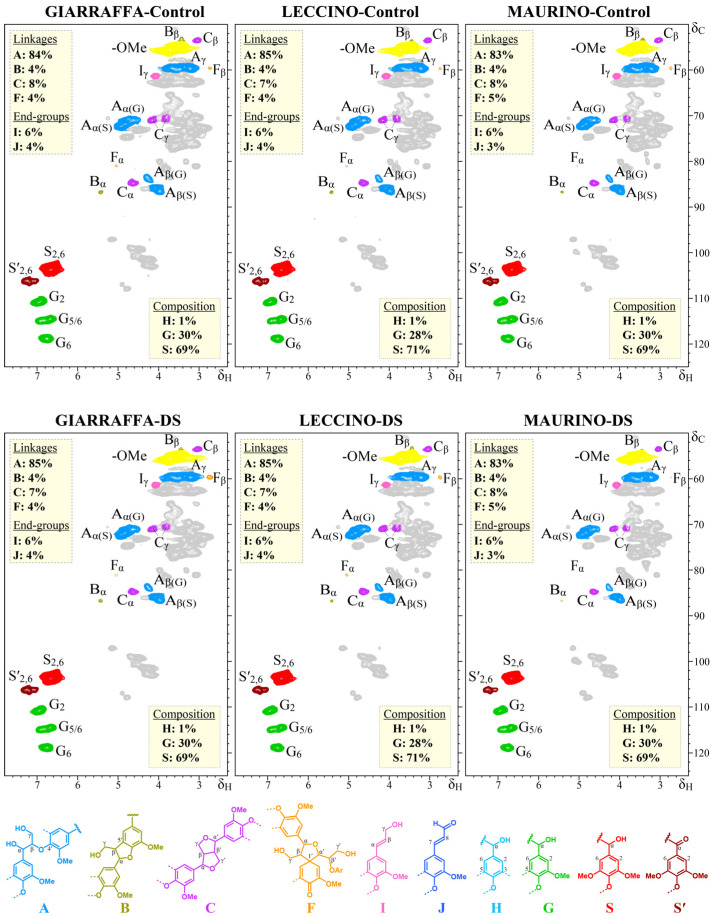
2D-HSQC NMR spectra of stems from three olive cultivars (Giarraffa, Leccino, and Maurino) subjected to drought stress (DS) (bottom) and their corresponding stem controls (top). The primary lignin structures identified are also shown. A: β-*O*-4′ alkyl-aryl ethers; B: β-5′ phenylcoumarans; C: β-β′ resinols; F: β-1′-spirodienones Cinnamyl alcohol end-groups (I), cinnamaldehyde end-groups (J), *p*-hydroxyphenyl units (H), guaiacyl units (G), syringyl units (S), and Cα-oxidized syringyl units (Sʹ). The yellow boxes reflect semi-quantitative estimates of lignin units and compounds. Composition is expressed in molar percent (H + G + S = 100%), and end-groups are expressed as a fraction of the total lignin inter-unit linkage types A–F.

Figure 8 shows that the HSQC spectra (δ_C_/δ_H_ 50–125/2.4–7.8) of all experimental groups revealed signals from carbohydrates (gray color) and lignin, as indicated by aromatic rings from guaiacyl (G) and syringyl (S) lignin units, including Cα-oxidized S-lignin units (S′). After increasing the threshold, only a weak signal for *p*-hydroxyphenyl (H) units was detected. In the aliphatic-oxygenated region of the spectra, typical signals from the Cα/Hα, Cβ/Hβ, and Cγ/Hγ correlations of the different interunit linkages (β-*O*-4′ alkyl-aryl ethers, A; β-5′ phenylcoumarans, B; β-β′ resinols, C; β-1′ spirodienones, F) and cinnamyl alcohol (I) end-groups were observed. 

A semi-quantitative estimation of the main structural characteristics of the lignins of the three olive cultivars, such as the relative abundances of the different inter-unit linkages (A, B, C, F), and cinnamyl end-groups (I, J), and the relative abundances of the lignin units (H, G, and S), estimated from the volume integration of the signals in the HSQC spectra, are indicated in the insets of the spectra in Figure 8. The lignin composition and structure were rather similar among the three olive cultivars. ‘Leccino’ lignin exhibited a slightly higher number of S-units, resulting in a higher S/G ratio (2.54) compared to ‘Giarraffa’ and ‘Maurino’ lignin (S/G ratio of 2.30). All three cultivars had similar amounts of inter-unit linkages and cinnamyl end-groups, with the majority being β-*O*-4′ alkyl-aryl ethers (83–85% of all linkages), followed by β-5′ phenylcoumarans (4%), β-β′ resinols (7–8%), and β-1′ spirodienones (4–5%). Cinnamyl alcohol and cinnamaldehyde end-groups constituted 6% and 3–4% of the total side chains, respectively. Overall, the data showed that the lignin content and structure in stressed stems of all cultivars were comparable to those in controls, indicating that drought stress does not affect lignin composition or structure in olive stems at this stage of development.

### 2.8. Xylem Anatomy

**Figure 9:**
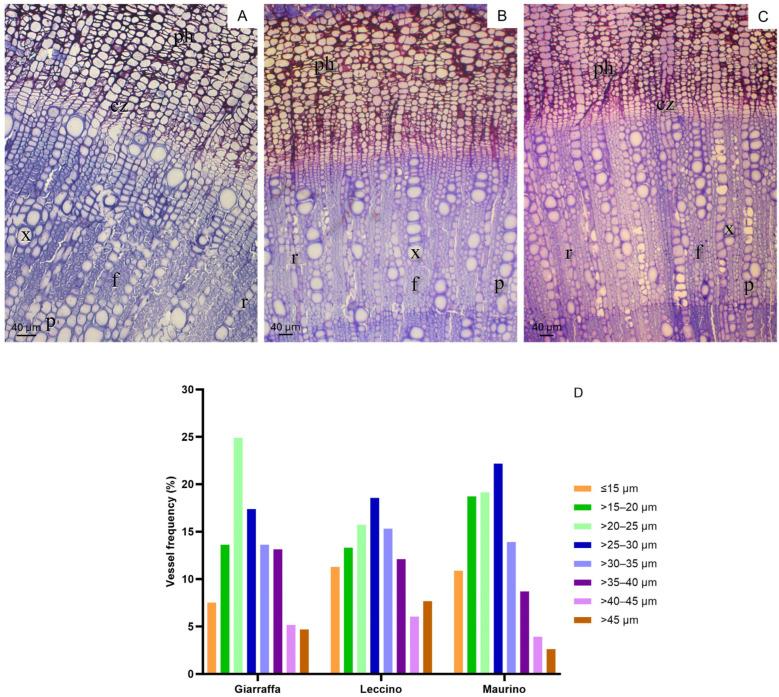
Stem sections of *Olea europaea* cultivars Giarraffa (**A**), Leccino (**B**), and Maurino (**C**). ph: phloem, x: xylem vessels; cz: cambial zone; r: parenchyma ray; f: fibers; p: paratracheal parenchyma; bar corresponds to 20 µm. (**D**) Frequency distributions (number of vessels by 5 µm diameter) of vessel lumen diameters in the three olive cultivars.

Figure 9 illustrates cross sections of the phloem (ph), cambial zone (cz) with thin cell walls and short radial diameters, and xylem. The xylem region comprises parenchyma rays (r), fibers (f) with thick cell walls and very small lumina, and paratracheal parenchyma (p), which resembles xylem but is smaller and exhibits a hexagonal cell wall under polarized light. Xylem vessels are typically observed in clusters of two or three cells.

Following data analysis, Figure 9B illustrates the distribution of xylem diameters categorized into 5 µm classes. Few vessels exceeded a diameter of 40 µm, with the most common frequencies ranging between 20 and 35 µm. In ‘Giarraffa’, many vessels had diameters of 20–25 µm, whereas in ‘Leccino’ and ‘Maurino’, the 25–30 µm class was predominant. Compared to ‘Giarraffa’, ‘Leccino’ and ‘Maurino’ exhibited smaller vessels (10–15 µm in diameter). However, no significant differences were observed between the cultivars. Table 1 presents the morphological and hydraulic data collected during the cross-section analysis. The cultivars did not differ significantly in vessel density, but ‘Maurino’ had more vessels per unit area than ‘Leccino’ and ‘Giarraffa’. Additionally, ‘Maurino’ displayed smaller average vessel diameters than the other two cultivars. The lumina of the vessels occupied a greater proportion of the xylem area in ‘Maurino’ compared to ‘Giarraffa’, which also had the lowest hydraulic diameter (in contrast to ‘Leccino’). The theoretical hydraulic conductivity did not differ significantly, but was lower in ‘Giarraffa’, higher in ‘Maurino’, and intermediate in ‘Leccino’.

## 3. Discussion

The aim of this study was to elucidate the mechanisms underlying the different responses to drought stress observed in three Italian olive cultivars: Giarraffa, Leccino and Maurino. Since previous research has consistently demonstrated cultivar-specific differences in drought tolerance, to gain a deeper understanding of these contrasting responses, we focused on compounds involved in water management, such as aquaporins, osmotins, dehydrins, sugars (including mannitol) and proline, and we studied the anatomical and chemical properties of the wood of the three cultivars. Overall, the results distinguished the cultivars and allowed them to be contextualized in a more comprehensive characterization of their drought-response strategy.

Sugars are a crucial class of metabolites in drought response. Primarily produced after photosynthesis, they provide energy for plant growth, serve as building blocks for complex polysaccharides, and act as osmoprotective solutes to maintain cell turgor under various abiotic stresses [8]. Drought can influence carbohydrate accumulation by altering the expression of specific genes in plant cells, such as those encoding sucrose synthase, invertase, hexokinase, and fructokinase. Consequently, regulating soluble carbohydrate concentrations is essential for plant adaptation to water scarcity [54]. Glucose was the predominant sugar in the leaves of the three cultivars. However, drought stress led to a reduction in glucose levels in both stems and leaves of all cultivars at t4. This indicates that a prolonged water deficit is required to cause a statistically significant decrease in glucose levels in drought-stressed samples of all olive cultivars. In plant cells, glucose serves several functions, including (1) storage as starch, (2) cellular respiration, and (3) conversion to other sugars [55]. In this study, starch content was analyzed, but no significant accumulation was observed, leading to the rejection of hypothesis 1 (conversion of glucose to starch). Although cellular respiration data were not collected, the use of glucose to enhance cellular respiration under drought stress (hypothesis 2) might offer benefits such as oxygen consumption and the removal of additional redox equivalents from chloroplasts [56]. Additionally, a decrease in glucose and an accumulation of fructose were detected in drought-stressed leaves, particularly in the Giarraffa cultivar at t4. The increase in fructose (hypothesis 3, conversion of glucose to other sugars) could be utilized to produce secondary metabolites like lignin and phenolic compounds [57]. Piccini et al. (2020) [58] observed a similar pattern of sugar accumulation in olive leaves exposed to UV-B radiation. 

In plant cells, sucrose can be broken down by either sucrose synthase or invertase, resulting in fructose + UDP-glucose or fructose + glucose, respectively. Sucrose synthase conserves energy during degradation, making it the preferred enzyme in stressful conditions, while invertase is less energy-efficient and is used when ATP is primarily needed [59]. The three olive cultivars showed different responses to drought in terms of sucrose accumulation: only the stressed groups of ‘Leccino’ and ‘Maurino’ exhibited increased sucrose levels in the leaf (at t4), whereas a significant increase was observed only in the stem of ‘Maurino’ (at t2). Sucrose, the primary transportable form of photosynthetic products, was found to be more abundant in cultivars that maintained stomatal conductance and carbon assimilation for longer periods during drought. Known as an anti-stress chemical compound, sucrose helps stabilize membranes and macromolecules in leaves [60]. In the stem, increased sucrose content in parenchyma cells and the apoplast may enhance the tissue’s water-storage capacity, maintain xylem transport, and facilitate embolism removal by refilling water-deprived xylem vessels [61,62]. Therefore, the substantial accumulation of sucrose in the stem of drought-stressed ‘Maurino’ could be seen as an attempt to counteract cavitation in plants not prone to stomatal closure [41].

Fructose serves as a precursor to mannitol [63], a polyol present in olive trees and other plants like celery and carrots. Mannitol aids cells in regulating osmotic potential, maintaining turgor, and protecting macromolecules from ROS [63]. At t4, all cultivars exhibited increased mannitol levels in their leaves and stems. Notably, in the stems of ‘Giarraffa’, mannitol began to accumulate as early as t2, likely aiding in water conservation in this plant organ. In contrast, mannitol levels in the stem of ‘Leccino’ decreased at t4. This may be linked to the fact that ‘Leccino’ consistently utilized another osmoprotectant, proline, which accumulated in both leaves and stems at t2. Proline may serve a similar role to mannitol in ‘Giarraffa’, enabling the ‘Leccino’ cultivar to maintain higher water content in both stem and leaf compared to ‘Maurino’. Increased levels of mannitol were found in the Tunisian cultivar Picholine, while the cultivar Meski experienced an increase in the total carbohydrate content in its wood [10]. The drought-resistant cultivar Chemlali also had a higher concentration of mannitol compared to the more susceptible cultivar Meski [15]. The Chemlali cultivar also maintained a higher proline content [9], along with the Zalmati cultivar [22].

Drought-stressed stems of ‘Leccino’ also accumulated a significant amount of PIP1 aquaporins, whereas the Maurino cultivar exhibited lower aquaporin content compared to the control. Conversely, ‘Giarraffa’ had a higher PIP1 content at t0, which decreased at subsequent time points. Overexpression of aquaporins in xylem-associated living cells was found to facilitate water flow (through the osmotic gradient) towards embolized vessels [61,62]. On the other hand, the accumulation of aquaporins may be related to the increase in stem conductivity observed during drought stress [64], as changes in hydraulic conductivity seem to be specifically regulated by PIPs [65]. Therefore, a decrease in PIPs combined with early stomatal closure after drought likely helps ‘Giarraffa’ retain water more efficiently. On the other hand, ‘Leccino’ closes its stomata later and requires water to sustain photosynthesis; the higher levels of aquaporins may provide the necessary water for photosynthesis, albeit at the cost of increased water consumption. Most studies on aquaporins and dehydration have focused on their expression in leaves and roots, and have found a stress-induced regulation of PIPs in these organs [37,66,67] and a likely association of PIPs with a decrease in physiological variables in poplar [68]. However, we have not yet found a clear relationship between physiological parameters such as leaf and stem water content analyzed in Parri et al. (2023) [51] and the different accumulation of aquaporins in the stems of the three cultivars; given the lack of data on the distribution of aquaporins in stems, we can only speculate about a relationship with the phenomenon of embolized xylem vessel refilling.

Although ‘Giarraffa’ showed an early increase in dehydrins in stressed stems, these proteins were only detectable in stressed leaves of ‘Maurino’ and ‘Leccino’. During drought exposure, dehydrins specifically act to reduce oxidative damage (such as malondialdehyde levels and electrolyte leakage) and increase water-holding capacity by accumulating appropriate solutes [69]. Dehydrins have been identified in olive trees as a response to cold treatment [28,33]. Since cold and drought stress share many genetic- and biochemical-response pathways, it was expected that osmotin levels would differ between watered and drought-stressed olive plants. A dehydrin gene isolated from wild olive trees has been shown to enhance drought tolerance in both wild olive and transgenic Arabidopsis plants [70]. This suggests that dehydrins may play a role in the drought-tolerance mechanism of olive plants. The consistent accumulation of dehydrins in ‘Maurino’ leaves may be interpreted as protection against potential oxidative damage, and may be related to its high susceptibility to electrolyte leakage. Furthermore, both ‘Leccino’ and ‘Maurino’ showed a prolonged maintenance of transpiration rate compared to ‘Giarraffa’, suggesting that the accumulation of dehydrins could be an effort to conserve water during high transpiration rates under drought conditions. Higher levels of dehydrins were also associated with drought susceptibility in the study by Conti et al. (2022) [71], where only the most drought-sensitive of four tomato cultivars accumulated dehydrins.

In contrast to dehydrins, osmotins seem to be more ubiquitous in the three cultivars, as they were found in both stems and leaves in all experimental groups. This protein appears to be a delayed response to drought, as it increased consistently only at t4, in both stems and leaves. Only in the stems of ‘Giarraffa’ did the osmotin levels not increase in drought-stressed samples compared to controls. Research on olive osmotin [35] and the data in this manuscript suggest that increased expression of the osmotin gene is a strategy used by olive trees to increase drought tolerance. Indirect support for this is provided by evidence that olive plants expressing the tobacco osmotin gene exhibited greater tolerance to drought stress [35]. However, in this case, the accumulation of dehydrins and osmotins may be more related to the “occurrence of drought stress” than to tolerance, since the most “drought-avoidant” cultivar, Giarraffa, invests less in protein accumulation, while ‘Leccino’ and ‘Maurino’ showed a similar response, although they had different oxidative-stress status.

In addition to biochemical responses, the chemical and morphological characteristics of olive wood are crucial, as they influence water flow along the stem and susceptibility to drought-induced cavitation [5,47]. A lignin content of about 23% of the total wood composition found in this study was also observed in branches from an olive grove in Spain [72] and in branches of the Spanish cultivar Picual [73]. In Rencoret et al. (2019) [73], olive tree branches had a lignin H:S:G composition of 2:48:50 with an S/G ratio of around 1. This was different from what was found in the samples of this study (H:G:S of around 1:30:69 and S/G ratio of 2.4). This discrepancy can be explained by the different age of the samples: branches from mature trees in the first case, young olive stems in the second. The values remained largely consistent across the cultivars, with ‘Leccino’ showing only slight variations. Notably, the drought-stressed stem of ‘Leccino’ exhibited a reduced lignin concentration, regardless of treatment. Generally, ‘Leccino’ had a higher composition of S monomers, leading to a greater S:G ratio compared to the other cultivars. The potential weakening of the conduit due to lower lignin content might have been offset by the increased S:G ratio, which enhances resistance to emboli [5]. Regarding drought stress, there was no significant difference in stem anatomy between control and stressed plants, likely because the xylem samples were collected on the final day of the study (30 days). In comparison, *Picea mariana* needs around 30 days for cell expansion and secondary cell-wall deposition [74]. Consequently, only a single ring of xylem cells was fully developed at the onset of water deficit, rendering changes from the controls undetectable. Thus, these analyses can be used to address characteristics in wood properties based on genotypic differences between cultivars, regardless of treatment. Anatomical studies were conducted on pooled samples from both control and stressed groups. Unlike their chemical properties, the three cultivars exhibited anatomical differences. The frequency distribution revealed that only a few vessels exceeded 15 µm in diameter, while ‘Leccino’ and ‘Maurino’ had a higher number of small vessels (≤15 µm), consistent with findings by Sabella et al. (2019) [49] and Walker et al. (2023) [75]. Morphologically (VD, d_m_ and A_v_), ‘Maurino’ displayed a higher vessel density and smaller average vessel diameter, resulting in a greater percentage of xylem vessel area per tissue area compared to the other two cultivars. Theoretical hydraulic conductivity (K_st_) is a crucial measurement in water balance studies, due to its negative correlation with whole-plant water-flow resistance. Increased K_st_ can result from larger vessel diameter and density [76]. Despite the anatomical characteristics of ‘Maurino’ indicating an increase in K_st_, no significant changes were observed. It can be inferred that the vascular density (VD) and average vessel diameter (A_v_) in ‘Maurino’, along with its high stomatal density, contribute to its higher stomatal conductance compared to other cultivars. Overall, it can be concluded that the vascular density (VD) and average vessel diameter (A_v_) in ‘Maurino’, together with its high stomatal density [51], create a conduit designed to maintain a high bulk of water flow from roots to leaves. This is reflected in the higher stomatal conductance found in the control samples of this cultivar compared to the others. However, ‘Maurino’ was found to be susceptible to higher water loss when exposed to drought, when considering only the stem water content, which was lower compared to the stressed samples of the other cultivars. No differences were found in the analyses of relative leaf water content. In order to maintain water in the leaf despite the high transpiration rate, this cultivar had to invest in water-related proteins, as suggested by the levels of dehydrins and osmotins. Conversely, ‘Giarraffa’ showed anatomical characteristics (low A_v_, VD and stomatal density) adapted to the management of lower water-flow availability and an investment in osmoprotectants greater in stems than in leaves, where it actually stored more water than the other cultivars when exposed to drought. ‘Leccino’ has intermediate characteristics, balancing water loss with osmoprotectant investment during stress.

## 4. Materials and Methods

### 4.1. Cultivation of Olive Plants and Application of Drought Stress

Growth conditions and stress application are described in Parri et al. (2023) [54]. Briefly, 18-month-old olive plants (*Olea europaea* L., cultivars Leccino, Maurino and Giarraffa) provided by “Spoolivi” (Società Pesciatina di Orticoltura, Pescia, PT, Italy) were grown in a 1:1 peat/pumice substrate [77] in a growth chamber with LED illumination (TLED secret Jardin—SRL AGOMOON, Manage, Belgium; light–dark cycle set to 12 h each). After acclimation with constant watering, 30 plants of each cultivar were equally divided into two groups: a control group (CTRL) and a drought-stress group (DS). The CTRL groups were fully watered, while the DS groups were fully water-deprived for 4 weeks. The drought period was divided into five time points (t0, t1, t2, t3, and t4), with t0 corresponding to the beginning of withholding irrigation and t1–t4 corresponding to the first, second, third, and fourth weeks of water deprivation. At t0, three plants per cultivar were sacrificed for stem analysis. At both time points, t2 and t4, three plants from each of the drought-stressed and control groups were sacrificed for stem analysis. Leaf samples were pooled from at least 6 individuals at all time points. A temperature and humidity logger provided an average temperature of 27.5 °C and an average humidity of 51.1%. The results of physiological analyses such as soil water content, leaf and stem relative water content and stomatal conductance, which assessed the effects of water deprivation on each experimental group, are reported in Parri et al. (2023) [51] and showed that from t2 onwards, the DS groups of all cultivars effectively experienced drought stress.

### 4.2. Extraction of Protein from Sampled Olive Stems and Leaves

All experimental groups were sampled at t0, t2 and t4. We excluded t0 from leaf protein analysis but extracted proteins from all other time points and relied on a comparison of drought-stressed and control samples at each time point. Because olive tissues are recalcitrant to protein extraction, we partially modified the Wu et al. (2014) method [78]. Three samples of 0.3 g stem/leaf powder were prepared for each experimental group. After vortexing for 30 s, 1.8 mL of 10% TCA/acetone and 20 μL of DTT were added to each sample. The samples were then stored at −20 °C for 20 min. After centrifugation (15,000 rcf, 5 min, 4 °C), the supernatants were discarded, and the pellets were washed three times with 80% acetone. After the final wash, the pellets were left at room temperature under vacuum to allow evaporation of solvents. Then, 0.8 mL SDS extraction buffer, 8 μL DTT 1 M, and 0.8 μL protease inhibitors were added to resuspend the pellet proteins. Samples were allowed to stand for 1 h at room temperature, with gentle agitation. After centrifugation (15,000 rcf, 10 min, RT), the supernatants were collected and divided into Eppendorf tubes (0.8 mL of supernatant per tube) and an identical volume of Tris-buffered phenol was added to each tube. Samples were vortexed for 3 min and then centrifuged at 15,000 rcf, 5 min, RT. The phenolic upper phases were collected in new tubes (max 0.4 each) to which 1.5 mL of ammonium acetate in methanol was added; samples were vortexed briefly, and left at −20 °C for 2 h. After centrifugation (15,000 rcf, 10 min, 4 °C), the supernatants were discarded. The pellets were washed twice with 80% acetone and held under vacuum for 2/3 min. To completely resuspend all pellets from the same experimental groups, 100 μL of Laemmli buffer was added and the mixture was heated at 95 °C for 5 min.

Due to potentially interfering substances in olive leaf and stem extracts, we have found that traditional methods for quantifying protein extracts, such as the Bradford Assay (Bio-Rad) or the 2-D Quant Kit (GE Healthcare), provide an inaccurate assessment of protein content. To improve protein quantification of samples and to address these issues, we used two different gel imaging systems, the Fluor-S and the Chemi-Doc (both from Bio-Rad); the choice of system depended on their respective availability at different times during the research. Specifically, the Chemi-Doc utilized Bio-Rad’s Stain-Free technology, which eliminated the need for accurate protein quantification prior to gel loading.

### 4.3. Electrophoresis and Immunoblotting of Leaf Proteins without Stain-Free Technology 

All protein extracts were separated in parallel with unstained molecular weight markers (Bio-Rad) on 10% bis-tris SDS-PAGE [79] at pH 6.5–6.8. Using XT MOPS (Bio-Rad Laboratories, Hercules, CA, USA) as the running buffer, and the run was approximately 45 min at 200 V. ImageJ (https://imagej.net/ij/index.html, accessed on 13 October 2024) was used to quantify proteins in each lane. To determine the loading volume of protein used for immunoblotting, a normalization factor was calculated for each extract. A second electrophoresis was performed using the normalized volume of the extracts and all-blue molecular-weight markers (Bio-Rad). Proteins were transferred to nitrocellulose membranes using the Trans-Blot Turbo Transfer System (Bio-Rad Laboratories, Milan, Italy) at 1.3 A and 25 V for 7 min. Membranes were incubated for 1 h in 0.1% Bio-Rad Tween 20 and 5% Bio-Rad Blotting-Grade Blocker solution in Tris-buffered saline (TBS). The membranes were washed twice with TBS for five minutes each time. Primary antibodies against osmotin (Agrisera AS194336 rabbit polyclonal, 1:1000, Vännäs, Sweden), dehydrins (Agrisera AS07206A rabbit polyclonal, 1:1000), and aquaporins (Agrisera AS09489 rabbit polyclonal, 1:1000) were added after removal of TBS. After gentle agitation for 1 h, the membranes were washed twice with TBS. The membranes were then incubated with a goat anti-rabbit secondary antibody (Bio-Rad 1706515, 1:3000) for 1 h and then rinsed twice with TBS. Clarity Max Western ECL (Bio-Rad Laboratories, Hercules, CA, USA) was used to visualize the immunological reaction. For relative quantitative assessment of band intensity, images of the blots were captured using a Fluor-S device (Bio-Rad Laboratories, Milan, Italy) and the data were analyzed using Bio-Rad Quantity One software 4.6.8 (Bio-Rad, Hercules, CA, USA).

### 4.4. Electrophoresis and Immunoblotting of Stem Proteins with Stain-Free Technology

Proteins were separated on polyacrylamide gels prepared with the TGX Stain-Free FastCast Acrylamide Kit 12% (Bio-Rad). Unstained protein standards (Bio-Rad) were used as molecular weight reference. The run was performed at 200 V for 45 min using TGS (tris/glycine/SDS) running buffer (Bio-Rad). Finally, the gel was placed into the ChemiDoc MP Imaging System (Bio-Rad) and subjected to the Stain-Free Gel procedure (590/110 UV Trans) with auto optimal exposure for 45 s. For immunoblotting, proteins were transferred to a nitrocellulose membrane using the Trans-Blot Turbo device (Bio-Rad) at 1.3 A, 25 V, for 7 min. Membranes were visualized in ChemiDoc using the Stain-Free Blot command (auto-optimized exposure) to verify proper transfer, and were then placed in 5% Blotting-Grade Blocker solution (Bio-Rad), 0.1% Tween 20 (Bio-Rad) in Tris-buffered saline (TBS) for one hour. After two 5 min washes with TBS, primary antibodies anti-osmotin (Agrisera AS19 4336 rabbit polyclonal 1:1000), anti-dehydrins (Agrisera AS07206A rabbit polyclonal 1:1000), and anti-aquaporins (Agrisera AS09 489 rabbit polyclonal 1:1000) were added and incubated for 1 h with gentle shaking. The membranes were then washed twice with TBS for 10 min each time and the secondary antibody (goat anti-rabbit, IgG Star-Bright Blue 700, Bio-Rad, code 12004162, 1:2500) was added to the membranes for one hour. Finally, the membranes were placed into ChemiDoc, and total proteins were visualized by selecting Stain-Free Blot, Auto-Optimal Exposure. The immunological reaction was visualized using the StarBright B700 Blot command (auto optimal exposure). Quantification of immunoblot signals was performed using Bio-Rad Image Lab software 6.1, (Bio-Rad, Hercules, CA, USA). Each membrane was visualized in the Stain-Free Blot channel and the lane with the highest protein abundance was selected as the reference. This lane was used to normalize the proteins present in all other lanes of the same membrane. The intensity of the immunoblotting signal in each lane was then normalized according to the relative protein content.

### 4.5. Proline Analysis

The proline content was determined according to Khedr et al. (2003) [80], starting from 100 mg of frozen leaf and stem powder mixed with 1.5 mL of 3% sulfosalicylic acid. The extracts were incubated for 1 h at 100 °C with 2 mL glacial acetic acid and 2 mL acidic ninhydrin. The samples were immediately cooled on ice and 1 mL of toluene was added. The absorbance of the upper phase (toluene-containing chromophore) was read at 520 nm. Proline content was determined from a calibration curve for D-proline; five leaf or stem samples were taken for each experimental group. Results are mean ± standard errors.

### 4.6. Sugar Analysis by High-Performance Liquid Chromatography (HPLC)

We analyzed sugars (sucrose, fructose, and glucose) and mannitol in the leaves and stems of all experimental groups sampled at t0, t2, and t4. The exception was the stem samples of ‘Maurino’ at t4, which were excluded due to structural conditions resulting from drought stress that prevented a proper analysis of sugars. Sugars were quantified according to Piccini et al. (2020) [58], with some modifications: before the addition of distilled water, 0.2 g (fresh weight, FW) of stem and leaf powder were dried at 70 °C for 36 and 24 h to obtain the dry weight (DW) of stem and leaf samples, respectively. Each sample was extracted twice to ensure total extraction of the sugars. Analysis was performed on a Waters Sugar-Pak I ion exchange column (6.5 × 300 mm) at 90 °C. MilliQ water (pH 7) was used as the mobile phase at a flow rate of 0.5 mL/min. The total run time was 30 min, and each sugar was identified using a Waters 2410 refractive index detector by comparing retention times with those of the standards. The curve area of each peak was plotted against that of the standards for quantification, and the results were expressed as the mean ± standard error of three replicates in mg/g DW.

### 4.7. Analysis of Starch Content

Starch content was determined according to the method of Loppi et al. (2021) [81], starting from 100 mg (FW) of stem and leaf powder dried at 70 °C for 36 and 24 h to obtain the dry weight (DW). The absorbance of the samples was read at 605 nm with a UV-Vis spectrophotometer (UV-1280, Shimadzu, Kyoto, Japan), and starch was quantified using a calibration curve (0.4–0.0025 mg/mL) prepared with soluble starch (Sigma-Aldrich, Kyoto, Japan). Results are expressed as the mean ± standard error of three replicates in mg/g DW.

### 4.8. Determination of Lignin Content

Stem samples of the three cultivars, CTRL and DS, at t4, were freeze-dried and manually debarked with a cutter, and the corresponding xylems were preground in a Retsch MM400 mill (Retsch, Haan, Germany). The preground samples were then sequentially extracted twice with 80% ethanol and once with acetone, for 30 min each, in an ultrasonic bath, centrifuged, and the supernatant removed. The extractive-free samples were finely ball-milled in a Retsch PM-100 planetary mill (Retsch, Haan, Germany) with a 50 mL agate jar and agate balls (10 × 10 mm) at 600 rpm for 2 h (alternating 10 min pauses every 20 min of milling) for further analysis. The lignin content was determined following the protocol of Lu et al. (2021) [82]. Measurements were performed in triplicate.

### 4.9. Analysis of Lignin Composition Using 2D-NMR Spectroscopy

For the determination of the lignin composition and structure, whole cell walls of olive stems were analyzed by 2D-NMR at the “gel state”, according to the previously published method [83,84]. Briefly, approximately 60 mg of finely ground, extractive-free samples were swollen in 0.6 mL DMSO-*d*_6_. 2D HSQC NMR spectra were recorded at 300 K on a Bruker AVANCE III 500 MHz instrument equipped with a cryogenically cooled 5 mm TCI inverted gradient probe. The HSQC experiments were performed using Bruker standard pulse programs “hsqcetgpsisp2.2” with the following parameters: spectra were acquired from 10 to 0 ppm in F2 (^1^H) using 1000 data points with an acquisition time of 100 ms and an interscan delay of 1 s, and from 200 to 0 ppm in F1 (^13^C) using 256 increments of 32 scans, for a total experiment time of 2 h 34 min. The ^1^*J*_CH_ used was 145 Hz. The processing used a typical matched Gaussian apodization in 1H and a squared cosine bell in ^13^C. The central solvent peak was used as an internal reference (δ_C_/δ_H_ 39.5/2.49). 2D-NMR cross-signals were assigned by literature comparison [73,84]. The volume integrals of the HSQC correlation peaks were analyzed semiquantitatively, using Bruker’s Topspin 3.5 processing software. In the aromatic/unsaturated region, the correlation signals of H_2,6_, G_2_, and S_2,6_ were used to estimate the content of the respective H-, G-, and S-lignin units (since the H_2,6_ and S_2,6_ signals involve two proton–carbon pairs, their volume integrals were halved). The different inter-unit linkages were quantified by the volume integrals of the Aα, Bα, Cα, and Fα correlation signals, corresponding to chemically analogous C/H with similar ^1^*J*_CH_ coupling values. The relative abundance of cinnamyl alcohol end groups (I) was estimated by integrating the signal Iγ, whereas the abundance of cinnamaldehyde end groups (J) was determined by integrating the signal Jβ and comparing it to Iβ, as previously described [73].

### 4.10. Stem Anatomy Analyses

Three 1cm long stem samples were collected at 40 cm from the collars of two plants in each experimental group. The samples were cut into thin slices and immersed in 1.5 mL of 3% glutaraldehyde for two hours. The samples were then washed three times for 10 min each, with 2 mL of cacodylate buffer. Samples were then dehydrated with increasing concentrations of ethanol (10%, 30%, 50% 70% and pure ethanol). The samples were then immersed in 2 mL of alcohol–resin solution (Technovit 7100, Heraeus Kulzer, Hanau, Germany) and kept in the dark with slight agitation with increasing resin volume (3:1, 1:1, 1:3, pure resin). Finally, the specimens were embedded in resin [84]. Transverse sections were cut with a rotary microtome. Sections were stained with cresyl violet acetate (0.16% in water) [85]. Anatomical observation was performed with a light microscope Zeiss Axiophot equipped with AxioCam MRm camera and the AxioVision software 4.6. Approximately 16 images of control groups per cultivar of the first woody ring before the area of the differentiating cells of the cambium were analyzed using ImageJ’s “Analyze Particles” tool (size: 50—infinity; circularity: 0.00–1.00) to obtain lumen Feret diameters and area, and total xylem area. The following parameters were calculated for each image [86,87]:

− Vessel-diameter frequency distribution: number of vessels by group of 5 µm diameter of vessel lumen diameters.
− Vessel density (VD, n./mm^−2^): number of xylem vessels per area of the xylem.
− Vessel diameter (d_m_, µm): average of the xylem Feret diameters.
− Vessel area/xylem area (A_v_, %): percentage of vessel lumen area/xylem area ratio.
− Hydraulic weighted-vessel diameter (D_H_, µm), calculated as

(1) $$D_{H}={d_{m}}^{5}/{d_{m}}^{4}$$

− Theoretical specific xylem hydraulic conductivity (K_st_, kg s^−1^ m^−1^ MPa^−1^), calculated as

(2) $$K_{st}=\frac{\pi \cdot \rho }{128\cdot A_{image}\cdot \eta }\cdot \Sigma {d_{m}}^{4}$$

where ρ is the density of water (998.2 Kg m^−3^ at 20 °C), η is the viscosity of water (1.002 × 10^−9^ MPa s at 20 °C), A_image_ is the area of the analyzed image (m^2^) and d_m_ is the vessel diameter.

### 4.11. Statistical Analyses

The distributions of the data for glucose, fructose, sucrose, mannitol, proline and starch were checked for normality using the Shapiro–Wilk test; differences between the experimental groups were assessed by a two-way ANOVA followed by a Tukey’s post hoc test. These data analyses and graphs were performed using GraphPad Prism software 10. Differences in the frequency distribution of vessel lumen diameters of the three cultivars were assessed using the chi-squared test. Significant differences for lignin content, lignin composition, VD, d_m_, A_v_, D_H_, and K_st_ of the three cultivars were calculated using one-way ANOVA followed by Tukey’s HSD post hoc test. These analyses were carried out using R studio.

## 5. Conclusions

Biochemical and structural analyses reveal how the three olive cultivars responded differently to drought stress. All cultivars showed decreased glucose levels in stressed leaves and stems, likely due to the use in cellular respiration or conversion to other sugars like fructose and UDP-glucose. An increase in leaf mannitol was observed in all cultivars, with ‘Giarraffa’ preferentially accumulating mannitol, while ‘Leccino’ accumulated more proline in stressed stems. The increase in sucrose content and the accumulation of aquaporins in the stressed stems of ‘Leccino’ and ‘Maurino’ may be related to their efforts to remove embolisms by refilling the xylem vessels, which is crucial for maintaining water transport and overall plant health under stress conditions. Furthermore, the accumulation of dehydrins and osmotin occurred mainly in ‘Leccino’ and ‘Maurino’, which maintained higher gas exchange rates compared to ‘Giarraffa’, suggesting that these cultivars have developed different strategies to cope with stress, allowing them to maintain better physiological performance. Wood chemistry, particularly lignin content and composition, was not influenced by genotype or stress. However, ‘Maurino’ had a higher vessel-area-to-xylem-area ratio and vessel density, but a smaller average vessel diameter, while ‘Leccino’ had the largest hydraulic diameter. These structural adaptations in ‘Maurino’ and ‘Leccino’ highlight the importance of anatomical features in stress resistance. Taken together, these results suggest three different strategies: ‘Giarraffa’ follows a “drought avoidance” strategy, which consists of an effective and timely stomatal control in response to soil water deficit. This strategy appears to be beneficial during short periods of water shortage, as it preserves the plant for future recovery. However, long-term stress can have negative effects on carbon fixation. Drought-tolerant species can maintain a positive carbon balance by maintaining prolonged basal gas-exchange rates. This is made possible by the investment in osmoregulatory capacity, which increases turgor during severe dehydration [88]. This seems to be the case with the Leccino cultivar. Finally, ‘Maurino’ appears to be severely dehydrated, due to its high water consumption. These findings underscore the importance of an integrated approach to defining drought-tolerance strategies, rather than focusing on individual parameters.

## Figures and Tables

**Table 1:** Mean values (±standard error) of morphological and hydraulic characteristics in the three olive cultivars. VD: vessel density; d_m_: mean vessel diameter; A_v_: ratio of total vessel area to total xylem area; D_H_: hydraulic diameter; K_st_: theoretical hydraulic conductivity. Different letters indicate statistical significance (*p*-value < 0.05) according to Tukey’s multiple post hoc test.

Cultivar	Morphological Traits			Hydraulic Traits	
	VD (n. mm^−2^)	d_m_ (µm)	A_v_ (%)	D_H_ (µm)	K_st_ (kg s^−1^ m^−1^ MPa^−1^)
Giarraffa	211.1 ± 27.9	30.0 ± 0.8 ^a^	6.0 ± 0.7 ^b^	30.5 ± 1.7 ^b^	2.6 ± 0.4
Leccino	237.1 ± 32.4	28.7 ± 0.7 ^a^	8.2 ± 0.5 ^ab^	36.7 ± 1.4 ^a^	3.7 ± 0.3
Maurino	251.6 ± 30.3	26.1 ± 0.7 ^b^	8.7 ± 1.1 ^a^	32.3 ± 1.3 ^ab^	3.5 ± 0.6

## Data Availability

The raw data supporting the conclusions of this article will be made available by the authors on request.

## Supplementary Materials

The following supporting information can be downloaded at: https://www.mdpi.com/article/10.3390/ijms252011059/s1.
