# Supplementary material for: Unravelling Different Water Management Strategies in Three Olive Cultivars: The Role of Osmoprotectants, Proteins, and Wood Properties
Source: Int J Mol Sci. 2024 Oct 15;25(20):11059. doi: 10.3390/ijms252011059 (PMC11507519; doi:10.3390/ijms252011059)
Supplement: Supplementary file 1 [file ijms-25-11059-s001.zip › ijms-3218171-supplementary.pdf]

Supplementary Materials

Unravelling different water management strategies in three olive cultivars: the role of osmoprotectants, proteins, and wood properties

Sara Parri [1], Claudia Faleri [1], Marco Romi [1], José C. del Río [2], Jorge Rencoret [2], Maria Celeste Pereira Dias [3], Sara Anichini [1,4], Claudio Cantini [5] and Giampiero Cai [1,*]

Starch content

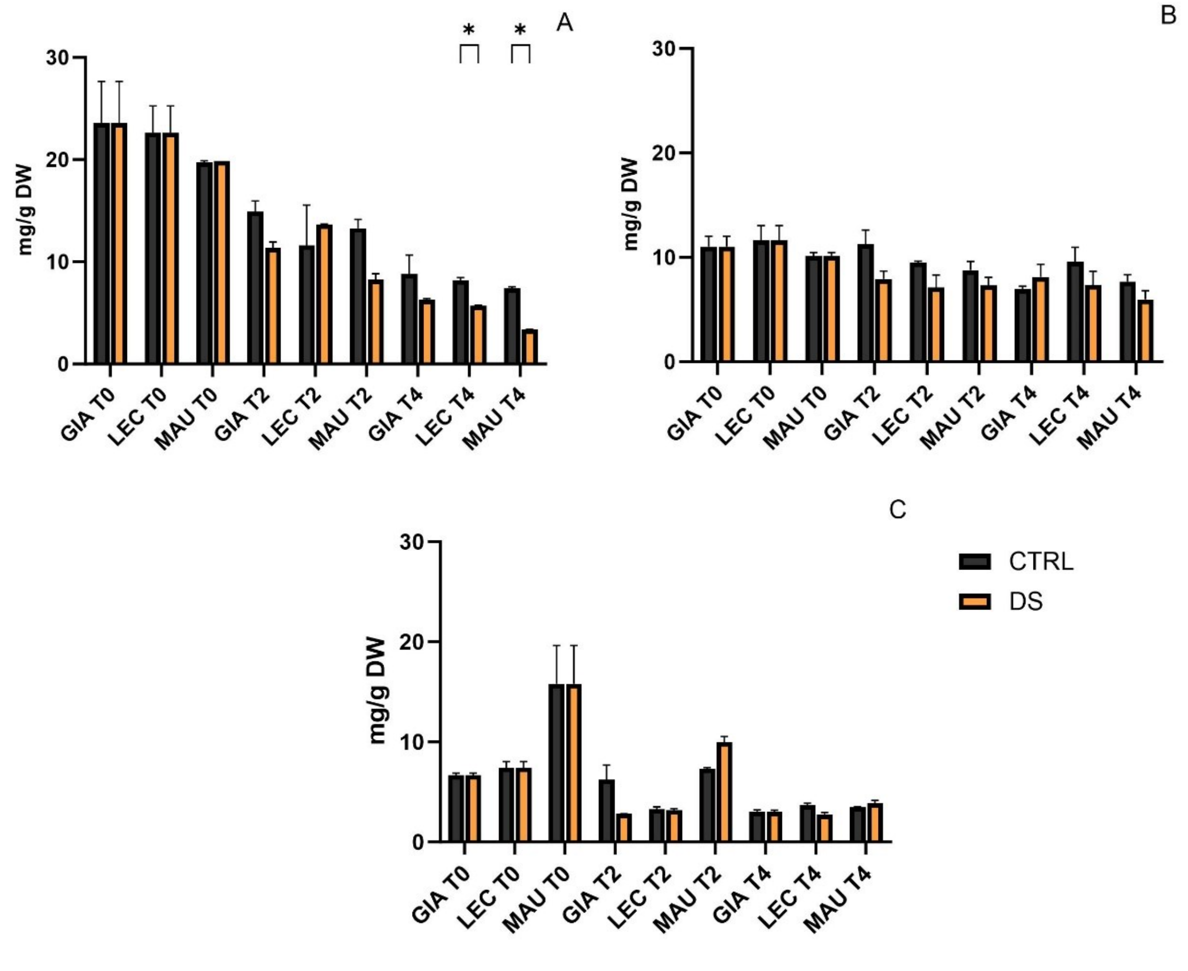

Figure S1. Starch content in leaves sampled at dawn (A), before night (B) and stem (C) of Giarraffa (GIA), Leccino (LEC) and Maurino (MAU) cultivars under control (CTRL, black) and drought stress (DS, orange) conditions. The contents are expressed as mg g^{-1} tissue dry weight (DW). For each column, values are expressed as mean ± standard error. Asterisks (*) indicate significant differences between control and treated groups (* = *p-value* < 0.05).
